# Osteoradionecrosis of the Temporal Bone Leading to Cerebellar Abscess

**DOI:** 10.1155/2017/7054943

**Published:** 2017-02-23

**Authors:** Thomas B. Layton

**Affiliations:** Faculty of Life Sciences, University of Manchester, Manchester M13 9PL, UK

## Abstract

Squamous cell carcinoma of the temporal bone is a rare and destructive malignancy and represents both diagnostic and therapeutic challenge. The complex regional anatomy of the temporal bone requires equally intricate surgical techniques to adequately resect the tumour mass during surgical excision. Adjuvant radiotherapy is offered to patients with advanced disease and has been showed to confer a survival benefit in carefully selected patients. One feared complication of radiotherapy is osteoradionecrosis and is a major obstacle faced in the treatment of head and neck cancers. The case presented here is a rare example of a patient who was successfully treated for SCC of the temporal with both surgical resection and adjuvant radiotherapy who subsequently developed two major complications: first, osteoradionecrosis of the temporal bone that leads to penetrating osteomyelitis; second, the formation of a large cerebellar abscess that required surgical drainage. This case is a rare example of the complications that are possible following radiotherapy to the head and the close follow-up that is required in patients.

## 1. Introduction

Squamous cell carcinoma (SCC) of the temporal bone was first described by Schwartze and Wilde in 1775, but it was not until 1917 when the first large scale review was published by Newhart [1, 2]. SCC of the temporal bone is a rare tumour with a reported incidence of between 1 and 6 cases per million population per year [3]. It accounts for less than 0.2% of all tumours of the head and neck but is the most common neoplasm in the external auditory canal [3]. SSC of the temporal bone is a tumour known for its relatively late and nebulous clinical presentation, as well as an aggressive tendency for local invasion [3]. Although strict risk factors for the disease have not been well established, chronic suppurative otitis media, cholesteatoma, and a history of radiation exposure have all been implicated [3]. Other putative risk factors are smoking and alcohol abuse. In a large study of patients diagnosed between 1945 and 2005 Gidley et al. (2009) stated that the mean patient age was 62 years (median, 63 years; range 21–89 years) and that the 5-year overall survival rate was 48% for early-stage disease and 28% for patients with late-stage disease [3]. In addition, Moffat et al. (2005) reported 80–100% five-year survival rates in patients who had successful primary surgical resection, with or without adjuvant radiotherapy [4].

In terms of management, a plethora of surgical techniques are available to combat the variable locations and sizes of the tumour mass: mastoidectomy, lateral and extended lateral temporal bone resection, and pinnectomy. The standard description is one of either standard or extended mastoidectomy with the later including a parotidectomy [5]. It has been suggested that a parotidectomy is mandatory for control of occult parotid node metastases and for optimizing adequate resection margins [5].

Adjuvant radiotherapy is offered to patients with locally advanced disease and has been shown to be beneficial [5]. The mean postoperative adjuvant radiotherapy given is 58.0 Gy (range, 29.6–75.0 Gy) to the primary tumour site and 49.5 Gy (range, 10.5–75.0 Gy) to the neck. Although radiotherapy offers a survival advantage in some well-selected patients it is not without the potential for significant morbidity [5]. One of feared sequelae of radiotherapy is radiation-induced bone injury, the most serious of which is osteoradionecrosis. This disease leads to the destruction of bone and by forming necrotic areas produces an ideal environment for bacterial colonisation and infection. It can also produce debilitating symptoms for patients including profuse and pulsatile otorrhoea and significant pain as well as leading to rare but potentially life threatening intracranial complication such as meningitis and abscess formation [6].

## 2. Case Report

A 50-year-old female presented with a four-month history right-sided headaches with occasional bloody otorrhoea. The patient had been treated with a mastoidectomy twenty years previous to chronic suppurative otitis media and a cholesteatoma but had no symptoms following her surgery. Past medical history included a schizophreniform disorder and Barrett's oesophagus under surveillance. Otoscopy revealed a mass within the middle ear and a biopsy confirmed moderately differentiated squamous cell carcinoma. A staging CT scan established that there was no distant metastatic disease and that the tumour was confined to the temporal bone. The TNM staging of the tumour was T3N2M0. A radical right mastoidectomy was performed that involved the removal of posterior and superior canal wall, meatoplasty, and exteriorisation of middle ear. At the time of surgery it appeared that the carcinoma was arising from the middle ear cleft and extending up to an eroded patch of tegmen. This was easily cleared but the eustachian tube orifice appeared widened and biopsy confirmed the presence of squamous carcinoma. In the hope of clearing residual disease the patient was offered adjuvant radiotherapy. The patient exhibited a good response to radiotherapy and showed a complete remission.

After treatment the patient was reviewed regularly at follow-up. Eleven months later the area of irradiated mastoid bone developed osteoradionecrosis (ORN) and periodically became infected. The initial diagnosis was made following a head and neck CT scan with intravenous contrast. The indication for imaging was a two- to three-week history of increasing pain adjacent to the surgical site and it was initially feared that this might have represented recurrent malignant disease. The radiological features included slight erosion and sclerosis of the adjacent temporal bone, no mass lesions, and mastoid opacification. It must be noted that the radiological features of ORN are often challenging to discriminate between malignancies, particularly in patients with a past history of cancer. The ORN was initially treated conservatively with regular local irrigation, analgesia, and antibiotics for episodes of infection. Repeated local cultures and blood cultures did not grow any organisms. After several months a CT head revealed a sequestrum in the temporal bone overlying the facial nerve and the patient suffered intermittent pain in the area of her ear that was treated with various analgesics including Tylex and amitriptyline. In addition a Tri-Adcortyl was inserted and Optomize was instilled into the external ear during episodes of infection. In addition, her mastoid cavity was periodically cleared through irrigation. The patient also suffered from recurrent episodes of labyrinthitis proposed to be caused by an exposed section of a semicircular canal adjacent to the sequestra of bone. These episodes were managed with vestibular sedatives and lasted only a few days. However, she did not suffer any significant hearing loss in either ear and between attacks of labyrinthitis her vestibular function was preserved without any associated symptoms. Stemetil was prescribed and these attacks became less and less frequent over the next 12 months.

Two years after the patient's initial surgery the area of necrotic temporal bone developed into penetrating osteomyelitis that lead to the formation of a 2 cm^3^ cerebellar abscess abutting the area of infected bone. The initial presentation was one of worsening headaches and an urgent MRI head revealed the presence of the abscess. In addition, the MRI revealed the sequestrum within the temporal bone opacification of the temporal bone adjacent to the abscess. No mass lesions were noted on imaging. The patient had no other features of raised intracranial pressure. The temporal bone osteomyelitis and cerebellar abscess were treated with broad-spectrum IV antibiotics and surgical drainage and irrigation through a burr hole. Local wound cultures and peripheral blood cultures were negative but local wound cultures grew fully sensitive* Pseudomonas aeruginosa*. The procedure was uneventful and the patient made a full recovery without any lasting neurological deficit.

At the time of writing the patient has repeated foul smelling otorrhoea. Repeated microsuction and toileting of the mastoid is performed during follow-up appointments every 3 to 6 months. Moreover, antibiotics are given when infections develop including Augmentin.

## 3. Discussion

Squamous cell carcinoma of the temporal bone is a rare and invasive tumour. Surgical resection is crucial as a treatment modality, and early surgical intervention is associated with an increased survival [3]. The patient presented here was treated with a radical mastoidectomy and adjuvant radiotherapy. At 17 years postoperatively there is no evidence of tumour recurrence so it appears that treatment was sufficient in controlling the disease. The plethora of surgical techniques available mandate a clear rationale when selecting a treatment strategy that aims to balance postoperative morbidity with adequate resection of the tumour.

An interesting feature of this care report is the patient's past history of chronic suppurative otitis media (CSOM) and previous mastoidectomy 13 years ago. Wierzbick et al. (2008) described a similar case in which a 67-year-old patient developed SCC in the postoperative cave 50 years after being operated on for a cholesteatoma [7]. CSOM has been proposed as a potential aetiological factor in middle ear cancer and the case here is an example that supports this hypothesis. The association between the two remains unclear and is yet to be studied in detail [8]. An important point is the need to consider any new symptoms such as severe earache and bleeding with suspicion in a patient with a history of CSOM. Early diagnosis of malignancy in such a case rests on a high index of suspicion and a thorough investigation that should always include multiple biopsies of the abnormal areas. Vikram et al. (2006) described three patients that had SCC of the middle ear with concurrent cholesteatoma and otitis media. This illustrates the importance of obtaining a sufficient histological specimen, as malignant cells can be found adjacent to area of both inflammation and infection [8].

SCC of the temporal bone is a tumour capable of significant local invasion and the use of adjuvant radiotherapy can lead to further weakening of the bone. The complexity of the anatomy of this region means that several delicate structures can be damaged including the middle and inner ear. This can produce debilitating symptoms for patients including hearing loss, vertigo, and tinnitus. The patient presented is a clear example of how disease complications arising from the temporal bone affected by radiotherapy can be significant morbidity. The patient suffered from recurrent episodes of severe labyrinthitis with one episode requiring hospital admission. Moreover, she developed recurrent headaches that were debilitating and significantly impacted upon her quality of life. She also has had multiple episodes of otitis and at present has a chronically discharging ear that requires repeated treatment. A challenge in managing patients with temporal bone SCC is balancing adequate surgical resection with the need for adjuvant radiotherapy. A more extensive surgical resection may reduce the need for radiotherapy but may also comprise the integrity of the remaining temporal bone structures. It is likely that surgical excision is likely to be tailored to an individual patient and will reflect not only the experience of the surgeon, but also the grade and stage of the tumour.

The most severe consequence of this was the development of osteomyelitis of the temporal bone that leads to the formation of a cerebellar abscess. Otogenic brain abscesses carry a high mortality rate and are one of many intracranial complications of osteoradionecrosis including meningitis, sigmoid sinus thrombosis, subdural empyema, perisinus abscess, and transverse and cavernous sinus thrombosis [6]. As part of the patient's follow-up it is paramount to screen for evidence not only of recurrence of the tumour, but also of any burgeoning infection and if detected antibiotics must be considered promptly.

## 4. Conclusion

Squamous cell carcinoma of the middle ear represents both diagnostic and surgical challenge and the case presented here provides a positive outcome with regard to the long-term survival following treatment. However, it also illustrates the significant impact that can follow this malignancy and its treatment. Osteoradionecrosis can have a great impact on a patient's well-being acutely with the potential for life threatening intracranial complications but also chronic symptoms that can severely hinder a patient's quality of life.
